# Decision Transformer-Based Efficient Data Offloading in LEO-IoT

**DOI:** 10.3390/e26100846

**Published:** 2024-10-07

**Authors:** Pengcheng Xia, Mengfei Zang, Jie Zhao, Ting Ma, Jie Zhang, Changxu Ni, Jun Li, Yiyang Ni

**Affiliations:** 1 School of Electronic and Optical Engineering, Nanjing University of Science and Technology, Nanjing 210094, China; pengcheng.xia@njust.edu.cn (P.X.); tingma@njust.edu.cn (T.M.); zhangjie666@njust.edu.cn (J.Z.); changxuni@njust.edu.cn (C.N.); jun.li@njust.edu.cn (J.L.); 2 Qian Xuesen College, Nanjing University of Science and Technology, Nanjing 210094, China; zangmengfei@njust.edu.cn; 3 School of Computer Engineering, Jiangsu Second Normal University, Nanjing 211200, China; niyy@jssnu.edu.cn

**Keywords:** LEO-IoT, data offloading, resource allocation, Decision Transformer

## Abstract

Recently, the Internet of Things (IoT) has witnessed rapid development. However, the scarcity of computing resources on the ground has constrained the application scenarios of IoT. Low Earth Orbit (LEO) satellites have drawn people’s attention due to their broader coverage and shorter transmission delay. They are capable of offloading more IoT computing tasks to mobile edge computing (MEC) servers with lower latency in order to address the issue of scarce computing resources on the ground. Nevertheless, it is highly challenging to share bandwidth and power resources among multiple IoT devices and LEO satellites. In this paper, we explore the efficient data offloading mechanism in the LEO satellite-based IoT (LEO-IoT), where LEO satellites forward data from the terrestrial to the MEC servers. Specifically, by optimally selecting the forwarding LEO satellite for each IoT task and allocating communication resources, we aim to minimize the data offloading latency and energy consumption. Particularly, we employ the state-of-the-art Decision Transformer (DT) to solve this optimization problem. We initially obtain a pre-trained DT through training on a specific task. Subsequently, the pre-trained DT is fine-tuned by acquiring a small quantity of data under the new task, enabling it to converge rapidly, with less training time and superior performance. Numerical simulation results demonstrate that in contrast to the classical reinforcement learning approach (Proximal Policy Optimization), the convergence speed of DT can be increased by up to three times, and the performance can be improved by up to 30%.

## 1. Introduction

The rapid proliferation of Internet of Things (IoT) devices has created enormous data processing demands, and traditional terrestrial network architectures face significant challenges in responding to these demands [1,2]. Mobile edge computing (MEC) servers can act as more powerful computation centers to help ground-based devices deal with some of the more complex computational issues [3,4,5]. Low Earth Orbit (LEO) satellites, by virtue of their near-Earth orbit characteristics, are capable of efficiently processing and transmitting data with low latency, thus relieving the pressure on ground-based networks [6,7,8]. Thus, the integration of LEO satellites with IoT networks and MEC systems provides a new way to address the growing demand for seamless and efficient communications [9,10,11]. In addition, the LEO satellite’s global coverage capability allows it to provide stable connectivity to remote areas, further expanding the boundaries of IoT applications.

Recent research has highlighted the significant role of LEO satellites in IoT communication systems. LEO satellites’ proximity to Earth allows for low-latency communication, which is critical for IoT applications requiring real-time data processing. Various studies have explored optimizing resource allocation, minimizing latency, and improving energy efficiency in LEO satellite-based systems. For instance, Jin et al. [12] analyze the development status of the IoT of the LEO satellite, put forward the architecture of the IoT of the LEO satellite, and analyze its characteristics to provide solutions and research directions for the in-depth study of the IoT of the LEO satellite. For narrowband IoT deployed on LEO satellites, Kodheli, O. et al. [13] propose a resource allocation method to reduce the high values of differential Doppler under the maximum value supported by the standard itself. Gao et al. [14] investigated the optimization of maximum completion time for IoT devices using non-orthogonal multiple access to transmit data to central Earth stations in LEO satellite–terrestrial integrated networks and for central Earth stations to transmit data to LEO satellites using orthogonal multiple access. They proposed a cooperative non-orthogonal multiple access scheme and, through matching theory, introduced a joint subcarrier assignment and cooperative non-orthogonal multiple access pairing method to minimize the maximum completion time in terrestrial networks. Additionally, they presented an iterative algorithm to optimize the maximum completion time among satellite beams. Fang et al. [15] explore the foundational models for integrating the fifth-generation technology with satellites in the sixth-generation networks to support widespread IoT applications. The authors propose an integrated satellite–terrestrial network architecture and analyze key technical challenges within the system, such as channel modeling, resource allocation, and network optimization. By establishing mathematical models and analyses, the article provides a theoretical basis for the seamless integration of satellite and terrestrial networks in future sixth-generation networks. Zhao et al. [16] consider an optimization problem of data transmission from numerous IoT terminals to LEO satellites and develop a scheduling algorithm to enable efficient data transmission, such that more IoT terminals can be connected and more data can be transmitted. Tondo et al. [17] explores optimal traffic load allocation for aloha-based IoT LEO constellations. They propose a novel traffic load distribution strategy based on successive convex approximation to maximize the system throughput. However, these optimization problems are typically NP-hard, and the computational cost is significant, especially in dynamic network environments with complex constraints, making real-time implementation challenging.

To address these challenges, recent research has increasingly turned to reinforcement learning methods [18]. Reinforcement learning offers distinct advantages in handling complex and dynamically changing systems by adaptively learning optimal strategies through interaction with the environment. For instance, Chai et al. [19] introduce an innovative bi-level deep reinforcement learning algorithm known as the Attention Mechanism and Proximal Policy Optimization Collaborative to optimize multitask offloading and resource allocation in satellite IoT environments, significantly reducing system costs and enhancing offloading efficiency. Furthermore, Han et al. [20], in response to the task offloading issue for remote IoT in integrated satellite–terrestrial networks, propose a two-timescale learning framework that employs a Hybrid Proximal Policy Optimization algorithm to effectively manage the dual-timescale network management decisions regarding task offloading link selection and bandwidth allocation. Lastly, Cao et al. [21] focus on LEO satellite-assisted multilayer MEC systems, devising an edge-assisted multilayer offloading optimization framework through the classic Alternating Optimization approach to provide low-energy and low-latency computing services. However, the direct use of reinforcement learning in wireless communication has some inherent drawbacks. First, reinforcement learning relies on extensive interactions with the environment, resulting in significant delays in achieving real-time responses and maintaining network efficiency. Second, reinforcement learning algorithms usually require a lot of training time to converge to an effective strategy. In addition, the most prominent shortcoming of reinforcement learning algorithms is the difficulty of generalization. That is, well-trained reinforcement learning strategies cannot be directly transferred to similar tasks. This limitation leads to a great waste of training resources.

To address the challenges posed by traditional reinforcement learning’s inefficiency in utilizing limited samples and adapting to new tasks, the development of Decision Transformer (DT) represents a significant advance [22,23,24]. DT takes advantage of Transformer’s advanced understanding and generalization features to handle complex decision problems. It turns reinforcement learning problems into serial modeling, and combined with the self-attention mechanism in Transformer, it can effectively combine previous decisions when making decisions. This structure allows it to further learn deeper and more varied task characteristics, thus making it more generalized. In simple terms, DT has the ability to generalize from pre-trained models and quickly adapt to new tasks through local fine-tuning.

This paper presents a system model in which LEO satellites act as information bridges between IoT users and a central MEC server. LEO satellites forward data from terrestrial to MEC servers to ease computing pressure on IoT devices. The model consists of IoT users, LEO satellites, a MEC server, and a ground-based decision center responsible for making offloading decisions. The main goal of the system is to reduce the delay and energy consumption during data transmission and computation by optimizing the resource allocation strategy. To solve the problem of resource allocation in such collaborative LEO-IoT systems, we first formulate resource allocation as an optimization problem. Then, the DT algorithm is used to solve this optimization problem and compare with Proximal Policy Optimization (PPO). Numerical simulation results demonstrate that the convergence speed of DT is augmented by over two to three times, and its performance is superior. The main contributions of this article are summarized as follows:

- We consider a collaborative LEO-IoT system with a ground information center to facilitate information sharing between LEO satellites and cooperation between LEOs, effectively solving the problem of insufficient IoT computing resources.
- We innovatively apply DT to solve resource optimization problems in the LEO-IoT data offloading scenario, which admits faster convergence and stronger generalization on new tasks than traditional reinforcement learning approaches.
- Our simulations demonstrate that DT architecture is superior to the benchmark algorithm. Specifically, the convergence speed of DT can be increased by up to three times, and the performance can be improved by up to 30% in the new scenario. This shows that DT can save a lot of time and computing resources in the application of new scenarios.

The remainder of this article is structured as follows: In Section 2, we formulate the resource allocation problem of the data offloading in LEO-IoT into an optimization problem. In Section 3, we transform the optimization problem into an MDP problem and propose the DT approach to solve it. In Section 4, we carry out extensive experiments with various settings to demonstrate that DT has stronger generalization and performance enhancements compared to traditional reinforcement learning algorithms. In Section 5, we conclude this paper.

## 2. System Model and, Furthermore, Problem Formulation

### 2.1. System Model

**Figure 1:**
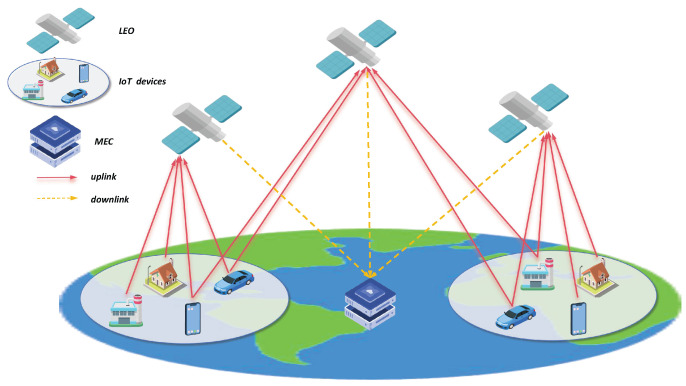
The data offloading of the LEO-IoT network.

In this paper, LEO satellites are employed as information bridges for users and the mobile edge computing (MEC) server, which is shown in Figure 1. We assume that there are *N* IoT users, and user *i* is denoted as $U_{i}$, where *i* belongs to the set I={1,...,N}. Supposing that there are *M* LEO satellites and only one MEC server, we denote the LEO satellite *j* as $L_{j}$, and *j* belongs to the set J={1,...,M}. At the same time, we add the Ground Decision Center to make the appropriate offloading decisions.

We assume that the computation task for each user is offloaded to the only MEC server via the LEO without local processing. After processing the data, the MEC server returns results to users. The offloading is operated in a time-slotted manner [25] within the total time period *T*. *T* is divided into *W* time slots, and each slot lasts for τ, where τ = *T*/*W* and *t* belongs to the set T={1,...,W}. In order to optimize the overall data offload, data are forwarded by the different satellites at different transmission rates in each time slot. Let $a_{ij}^{t}$ represent the association situation between the *i*-th user and *j*-th LEO:

(1) $$a_{ij}^{t}=\left\{\begin{array}{cc} 1, & U_{i}\;\mathrm{associate}\;\mathrm{with}\;L_{j},\forall i,j\in I,J,\\ 0, & U_{i}\;\mathrm{not}\;\mathrm{associate}\;\mathrm{with}\;L_{j},\forall i,j\in I,J. \end{array}\right.$$

Therefore, the transmission bandwidth and the transmit power for all uplinks and downlinks vary across different time slots. The transmit power for all uplinks is provided by users, and the transmit power for downlinks is provided by satellites. We assume that LEO satellites are working at Ka-Band [26] and the center frequency of uplinks and downlinks is 20 GHz.

At the beginning of each time slot, each user provides a workload summary to the Ground Decision Center. Based on this information, the Ground Decision Center decides on an offloading policy and returns the policy to the users and the LEO satellites. Only a small amount of allocated information (hundreds of bits) is transmitted during this process, and the communication lasts for a short period of time compared to the ensuing data (millions of bits) offload. We ignore the corresponding power and bandwidth consumption as well as the transmission delay of these communication links. In addition, the data are processed by the MEC server and the results are returned to the user by the LEO satellite. Since the number of computed results is much smaller than the number of data, we ignore the corresponding energy consumption and delay [27].

To allocate the bandwidth resource dynamically, the satellite assigns bandwidth to related links. The bandwidth for the link from $U_{i}$ to $L_{j}$ at t is denoted as $B_{ij}^{t}$, and that from $L_{j}$ to the MEC server is denoted as $B_{j}^{t}$. The transmit power of users is also allocated dynamically. The transmit power for the link from $U_{i}$ to $L_{j}$ at t is denoted as $P_{ij}^{t}$, and the power from $L_{j}$ to the MEC server corresponds to the maximum transmit power $P_{L}$.

### 2.2. Communication Model

#### 2.2.1. Uplink

For the uplink communications, the altitude, speed, and position information of the LEO satellites is known to the Ground Decision Center in a time slot due to the orbital pre-planning. For the sake of simplicity, a quasi-static fading channel model is considered in a time slot. Thus, the channel between $U_{i}$ and the LEOs is given by

(2) $$h_{ij}^{\mathrm{up}}=v_{ij}\gamma_{ij}{\hat{d}}_{ij}^{-\beta },$$

where $v_{ij}∼\left(0,1\right)$ is a complex Gaussian variable representing Rayleigh fading, $\gamma_{ij}$ follows log-normal distributed shadow fading, ${\hat{d}}_{ij}$ is the distance between $U_{i}$ and $L_{j}$, and β is the path loss exponent.

Thus, the signal-to-noise ratio can be obtained as

(3) $$SNR_{ij}^{t}=\frac{P_{ij}^{t}{\left|h_{ij}^{\mathrm{up}}\right|}^{2}}{N_{0}B_{ij}^{t}},$$

where $N_{0}$ is the power spectral density of the additive white Gaussian noise (AWGN). $N_{0}B_{ij}^{t}$ is the Gaussian white noise power for links from $U_{i}$ to $L_{j}$. $P_{ij}^{t}$ represents the transmit power for links from $U_{i}$ to $L_{j}$. According to the Shannon equation, the achievable capacity of the uplink is expressed as

(4) $$R_{ij}^{t}=B_{ij}^{t}\log_{2}\left(1+SNR_{ij}^{t}\right),$$

where $B_{ij}^{t}$ and is the allocated frequency resource for links from $U_{i}$ to $L_{j}$.

#### 2.2.2. Downlink

Different from the uplink case, the satellite channel generally experiences less fluctuation considering the line-of-sight link between the satellite and MEC. Thus, the channel model of the satellite can be considered as an additive white Gaussian noise (AWGN) channel [28]. According to the large-scale fading model in the literature [29], the channel between $L_{j}$ and the MEC server is given by [30]

(5) $$h_{j}^{\mathrm{down}}=\frac{\lambda^{\prime }G}{4\pi r^{sat}},$$

where $\lambda^{\prime }$ denotes the wave length, $r^{\mathrm{Sat}}$ denotes the distance between the MEC and satellite, and *G* is antenna gain. In the same way as the uplink is calculated, the signal-to-noise ratio of the downlink is denoted by

(6) $$SNR_{j}^{t}=\frac{P_{L}^{t}{\left|h_{j}^{\mathrm{down}}\right|}^{2}}{N_{0}B_{j}^{t}},$$

where $N_{0}$ is the power spectral density of the additive white Gaussian noise (AWGN). $N_{0}B_{j}^{t}$ is the Gaussian white noise power for links from $L_{j}$ to the MEC server. $P_{L}$ represents the transmit power for links from $L_{j}$ to the MEC server. According to the Shannon equation, the achievable capacity of the downlink is expressed as

(7) $$R_{j}^{t}=B_{j}^{t}\log_{2}\left(1+SNR_{j}^{t}\right),$$

where $B_{j}^{t}$ and is the allocated frequency resource for links from $L_{j}$ to the MEC server.

### 2.3. Delay Model

We assume that there is $V_{i}^{t}$ amount of data left to be transmitted at time slot *t* for $U_{i}$. For each time slot, part of the data will be transmitted and the amount of residual data will be changed. The data left at time slot t+1 can be calculated as

(8) $$V_{i}^{t+1}=V_{i}^{t}-\sum_{j=1}^{M}R_{ij}^{t}a_{ij}^{t}\min\left(\frac{V_{i}^{t}}{\sum_{j=1}^{M}R_{ij}^{t}a_{ij}^{t}},\tau \right),$$

where $\sum_{j=1}^{M}R_{ij}^{t}a_{ij}^{t}$ is the sum rate for all uplinks started from $U_{i}$ to satellites and τ is the time slot length. $\min\left(\frac{V_{i}^{t}}{\sum_{j=1}^{M}R_{ij}^{t}a_{ij}^{t}},\tau \right)$ represents the actual transmission time within each time slot.

So, the total delay of the uplink can be expressed as

(9) $$T_{\mathrm{up}}=\sum_{t=1}^{W}\sum_{i=1}^{N}\min\left(\frac{V_{i}^{t}}{\sum_{j=1}^{M}R_{ij}^{t}a_{ij}^{t}},\tau \right).$$

Since the satellite only serves as a bridge between the user and the MEC server, we assume that there is no data buildup on the satellite. That is, the downlink transmission rate of all satellites is greater than the sum of the rates of all their uplinks. So while the uplink transmission is completed, the downlink transmission is also completed at the same time. So we only need to consider the delay of the uplink.

### 2.4. Energy Model

We have calculated the transmission delay and combined it with the power and can derive the energy consumption of the uplink:

(10) $$E_{\mathrm{up}}=\sum_{t=1}^{W}\sum_{i=1}^{N}\left(\min\left(\frac{V_{i}^{t}}{\sum_{j=1}^{M}R_{ij}^{t}a_{ij}^{t}},\tau \right)\times \sum_{j=1}^{M}P_{ij}^{t}a_{ij}^{t}\right).$$

For downlink energy consumption, we define $t_{j}^{\max}$ to be equal to the longest transmission time among all uplinks of satellite $L_{j}$. Then $t_{j}^{\max}$ can be expressed as

(11) $$t_{j}^{\max}=\max_{i}\left(\min\left(\frac{V_{i}^{t}}{\sum_{j=1}^{M}R_{ij}^{t}a_{ij}^{t}},\tau \right)a_{ij}^{t}\right),$$

where *i* belongs to the set I and $a_{t}^{ij}$ is equal to 1.

So, the energy consumption of the downlink can be expressed as

(12) $$E_{\mathrm{down}}=\sum_{t=1}^{W}\sum_{j=1}^{M}t_{j}^{\max}P_{L}.$$

The total energy consumption can be expressed as the sum of the uplink and downlink energy consumption:

(13) $$E_{\mathrm{tot}}=E_{\mathrm{up}}+E_{\mathrm{down}}.$$

### 2.5. Problem Formulation

Based on the above models, we formulate the optimization problem as follows:

(14) $$\begin{array}{ccc} \left(P1\right) & \; & \min_{B_{ij}^{t},B_{j}^{t},P_{ij}^{t},a_{ij}^{t}}T_{\mathrm{up}}+\alpha E_{\mathrm{tot}} \end{array}$$

(15) $$\begin{array}{cc} s.t.\; & \sum_{j=1}^{M}P_{ij}^{t}a_{ij}^{t}\leq P_{U},\forall i\in I,\forall t\in T, \end{array}$$

(16) $$\begin{array}{cc} \; & \sum_{i=1}^{N}B_{ij}^{t}a_{ij}^{t}+B_{j}^{t}\leq B^{tot},\forall j\in J,\forall t\in T, \end{array}$$

(17) $$\begin{array}{cc} \; & \sum_{i=1}^{N}R_{ij}^{t}a_{ij}^{t}\leq R_{j}^{t},\forall i\in I,\forall t\in T, \end{array}$$

(18) $$\begin{array}{cc} \; & a_{ij}^{t}\in \left\{0,1\right\},\forall i\in I,\forall j\in J,\forall t\in T. \end{array}$$

The constraint in (15) indicates that the sum of the user’s transmit power should be less than the maximum power $P_{U}$. From the constraint in Equation (16), it can be seen that the sum of the uplink and downlink bandwidth resources corresponding to all LEO satellites is less than the sum of the available frequency resources of the satellite. The constraint in (17) means that since there is no buffer on the LEO satellite, the sum of the rates of the uplinks of the LEO satellite should be less than the sum of the rates of the downlinks, otherwise congestion will occur. α is used to balance latency and energy consumption.

## 3. Computation Offloading Scheme via Decision Transformer

In this section, we transform the optimization problem into an MDP problem and solve it with DT. The MDP problem can be defined as a tuple of (S,A,R). S denotes the set of global states, and $s^{t}$ is the state of time slot t. The agent takes an action $a^{t}$ at time slot *t*. A is the set of actions. The transition of the state is according to the function

(19) $$P_{s^{t},s^{t+1}}^{a^{t}}=P\left(s^{t+1}|s^{t},a^{t}\right):S\times A\to S,$$

where $s^{t+1}$ is the state at time slot t+1.

After performing $a^{t}$, the agent obtains the reward by the function

(20) $$R(s^{t},a^{t}):S\times A\to R,$$

where $r^{t}$ is the reward at time slot *t*.

To define the MDP more clearly, the state, action, and reward should be designed carefully. Details are described as follows.

### 3.1. Agent

The Ground Decision Center acts as an intelligence in the environment, allocating bandwidth and power resources for global uplinks and downlinks.

### 3.2. State

Our task is to offload computational tasks from the IoT device to the MEC server via LEO satellite. So our state at each moment can be expressed in terms of the task residual on the IoT device. We represent the state of each moment as

(21) $$s^{t}=\left(V_{1}^{t},V_{2}^{t},\cdots ,V_{N}^{t}\right),$$

where *N* indicates the number of users and $V_{i}^{t}$ indicates the amount of data left to be transmitted at time slot t for $U_{i}$.

### 3.3. Action

The specific action vectors of the terrestrial decision center can be divided into three vectors: (1) power vector, (2) bandwidth vector, and (3) selection vector. We represent each vector as an N×M matrix, where *N* represents the number of users and *M* represents the number of satellites. The values in the corresponding *i*-th row and *j*-th column of the matrix represent the decision values between the *i*-th user and the *j*-th satellite, i∈N,j∈M.

For the power resource, the Ground Decision Center needs to determine the power of all relevant uplinks, while the power of each downlink is fixed. We provide several reasonable values within the user’s power range for the Ground Decision Center to select. We represent the power vector at each moment as

(22) $$a_{P}^{t}=\left(\begin{array}{c} \begin{array}{ccc} P_{11}^{t} & \cdots & P_{1M}^{t}\\ \vdots & \ddots & \vdots \\ P_{N1}^{t} & \cdots & P_{NM}^{t} \end{array} \end{array}\right),$$

where *N* indicates the number of users and *M* the number of LEOs.

For bandwidth resources, the Ground Decision Center determines the frequency resources for all relevant uplinks. We provide several reasonable values within the satellite’s bandwidth range for the Ground Decision Center to choose from. Meanwhile, in order to make full use of the satellite’s bandwidth resources, the bandwidth of the downlink is equal to the total bandwidth minus the satellite’s uplink bandwidth, which can be expressed as $B_{j}^{t}=B^{tot}-\sum_{i=1}^{N}B_{ij}^{t}a_{ij}^{t}$. So, we represent the bandwidth vector at each moment as

(23) $$a_{B}^{t}=\left(\begin{array}{c} \begin{array}{ccc} B_{11}^{t} & \cdots & B_{1M}^{t}\\ \vdots & \ddots & \vdots \\ B_{N1}^{t} & \cdots & B_{NM}^{t} \end{array} \end{array}\right),$$

where *N* indicates the number of users and *M* the number of LEOs.

For the selection vector, we define it as a simple 01 selection, where 1 represents *i* and *j* connected and 0 represents them disconnected. We represent the selection vector at each moment as

(24) $$a_{S}^{t}=\left(\begin{array}{c} \begin{array}{ccc} a_{11}^{t} & \cdots & a_{1M}^{t}\\ \vdots & \ddots & \vdots \\ a_{N1}^{t} & \cdots & a_{NM}^{t} \end{array} \end{array}\right),$$

where *N* indicates the number of users and *M* the number of LEOs.

### 3.4. Reward

Our reward function is based on the final optimization goal and its constraints. In the following, we convert the latency $\sum_{t=1}^{W}\sum_{i=1}^{N}min\left(\frac{V_{i}^{t}}{\sum_{j=1}^{M}r_{ij}^{t}a_{ij}^{t}},\tau \right)$ during *T* to $r_{L}^{t}$ for each time slot *t*:

(25) $$r_{L}^{t}=\sum_{i=1}^{N}min\left(\frac{V_{i}^{t}}{\sum_{j=1}^{M}R_{ij}^{t}a_{ij}^{t}},\tau \right),$$

where smaller $r_{L}^{t}$ represents better allocation at time slot *t*.

The energy consumption of all users during *T* could be converted to $r_{EU}^{t}$ at time slot *t*, where

(26) $$r_{EU}^{t}=\sum_{i=1}^{N}\left(\min\left(\frac{V_{i}^{t}}{\sum_{j=1}^{M}R_{ij}^{t}a_{ij}^{t}},\tau \right)\times \sum_{j=1}^{M}P_{ij}^{t}a_{ij}^{t}\right).$$

The energy consumption of all LEO satellites is denoted as $r_{EL}^{t}$ at time slot *t*, where

(27) $$r_{EL}^{t}=\sum_{j=1}^{M}t_{j}^{\max}P_{L}.$$

The total energy consumption reward for both users and LEO satellites is

(28) $$r_{E}^{t}=r_{EU}^{t}+r_{EL}^{t},$$

where smaller $r_{E}^{t}$ represents better allocation at time slot *t*.

To satisfy the constraints in (15), we add a penalty term $r_{C1}^{t}$:

(29) $$r_{C1}^{t}=\sum_{i=1}^{N}\left(\sum_{j=1}^{M}P_{ij}^{t}a_{ij}^{t}-P_{U}\right),$$

where $r_{C1}^{t}$ is smaller, so the allocation is better.

To satisfy the constraints in (17), we add another penalty term $r_{C2}^{t}$:

(30) $$r_{C2}^{t}=\sum_{j=1}^{M}\left(\sum_{i=1}^{N}R_{ij}^{t}a_{ij}^{t}-R_{j}^{t}\right),$$

where $r_{C2}^{t}$ is smaller, so the allocation is better.

The total penalty term $r_{C}^{t}$ for constraints in (15) and (17) is

(31) $$r_{C}^{t}=r_{C1}^{t}+r_{C2}^{t}.$$

The constraints in (16) have been satisfied by the design of the action structure. Then the reward is

(32) $$r^{t}=-\left(r_{L}^{t}+\alpha r_{E}^{t}+\beta r_{C}^{t}\right),$$

where α is the coefficient for adjusting the optimization function in (P1) and β is the coefficient to satisfy constraints.

To date, the optimization problem with constraints has been transformed into Equation (32).

### 3.5. Decision Transformer

**Figure 2:**
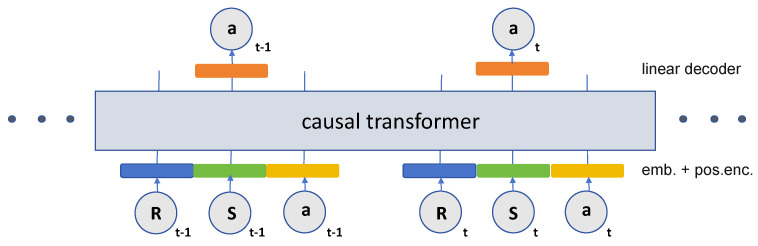
Decision Transformer model.

DT is an offline reinforcement learning model with the structure shown in Figure 2. We summarize the details of DT in Algorithm 1. It is an innovative approach to redefining reinforcement learning (RL) by applying sequence modeling techniques. Unlike traditional reinforcement learning approaches that rely on estimating value functions or calculating policy gradients, DT utilizes the Transformer architecture [31] to predict future actions based on sequences of past states, actions, and rewards. This sequence-based approach enables the model to capture long-term dependencies and complex patterns in trajectory data, making the learning process more flexible and with greater generalization capabilities. It can outperform traditional reinforcement learning algorithms in new scenarios by being pre-trained on large-scale datasets and subsequently fine-tuned on new specific tasks with minimal additional data [32]. This feature is particularly useful in wireless communications and IoT applications, where rapid adaptation to changing environments and efficient resource management are required. We summarize DT’s key features [24] in the following three points:

- In DT, decision making is treated as a sequence modeling task, where an agent’s interactions with its environment are depicted as a series of states, actions, and rewards: $\chi =\{\left(s_{1},a_{1},r_{1}\right),\left(s_{2},a_{2},r_{2}\right),\ldots ,\left(s_{T},a_{T},r_{T}\right)\}$. Here, $s_{t}$ denotes the state at time step *t*, which is the same as the $s^{t}$ we defined before. $a_{t}$ denotes the action taken at time step *t*, which is the same as the $a^{t}$ we defined before. $r^{t}$ and $r_{t}$ are different. $r_{t}$ denotes the cumulative expected return from the beginning of time step *t* to the end of the time step, which can be expressed as $r_{t}=\sum_{t=t}^{T}r^{t}$ (this emphasis on cumulative returns can help to improve policies effectively). The goal of sequence modeling is to predict future actions by learning the patterns of these sequences, thus enabling effective decision making by intelligences in their environment.
- DT is trained offline by generating data from other trained models interacting with the environment and can be deployed to new tasks with simple online fine-tuning.
- Unlike reinforcement learning models that only react to the current state, DT utilizes the entire sequence of historical information encoded with locations to predict future actions. This feature is particularly important in stochastic and dynamic environments, where the optimal action at any step may depend on a set of prior experiences and decisions.

**Algorithm 1** Decision Transformer.
**Input:** Offline data D : $\psi =\{\left(s_{1},a_{1},r_{1}\right),\left(s_{2},a_{2},r_{2}\right),\ldots ,\left(s_{T},a_{T},r_{T}\right)\}$. s is states. a is actions. r is reward.1:Initialize θ for the Causal transformer;2:Initialize context length *K*, batch size *B*, the learning rate μ;3:Data preprocessing D : $\psi =\{\left(R_{1},s_{1},a_{1}\right),\left(R_{2},s_{2},a_{2}\right),\ldots ,\left(R_{T},s_{T},a_{T}\right)\}$; R is returns -to -go;4:**Training phase**:5:**for** (R , s , a , t) in D **do**6:    Get the data with dimensions (B, K, dim), dim is the respective dimension of R, s, a and t;7:    Predicted action $a_{preds}$ = DecisionTransformer(R, s, a, t);8:    loss = mean(($a_{preds}$ - a)**2);9:    Update θ by loss;10:
**end for**
11:**Evaluation phase**:12:Set final target return: $target_{return}$13:Initialize environment parameters: R = $target_{return}$, s = env.reset(), a is null, t = 1, done = False14:**while not** done **do**15:    action = DecisionTransformer (R , s , a , t)16:    $s_{new}$, r, done ← env.step(action)17:    R, s, a, t ← R - r, $s_{new}$, action, t + length of R18:
**end while**



## 4. Numerical Simulation and Analysis

The PPO algorithm [33,34,35], which stands for Proximal Policy Optimization, is an efficient policy gradient reinforcement learning algorithm. It optimizes the policy by introducing a clipped probability ratio, ensuring that policy updates do not deviate too far from the current policy, thus avoiding significant performance fluctuations during the policy update process. The PPO algorithm uses a hyperparameter ϵ to control the magnitude of policy updates, keeping the updated policy close to the original policy in probability distribution, which helps achieve a more stable and reliable learning process. Additionally, the PPO algorithm employs two important techniques: it estimates the first and second moments of the policy gradient using multiple mini-batches of samples and balances exploration and exploitation through an adaptive objective function. These features make PPO exhibit excellent performance and stability across various tasks and environments. In this section, we use the parameter settings shown in Table 1 to evaluate the performance of PPO and DT in the environment we set up.

We designed a total of four sets of experiments, namely, one IoT device with two LEO satellites, two IoT devices with one LEO satellite, two IoT devices with two LEO satellites, and three IoT devices with two LEO satellites. We denote them as Experiment I, Experiment II, Experiment III and Experiment IV, respectively. The offline dataset used by DT for pre-training is derived from the traditional RL algorithm PPO. During the fine-tuning process, a small number of samples are generated by interacting with the environment of the new task using the PPO algorithm from scratch and using the small number of samples to precisely fine-tune the DT model, namely, DT-FT.

**Figure 3:**
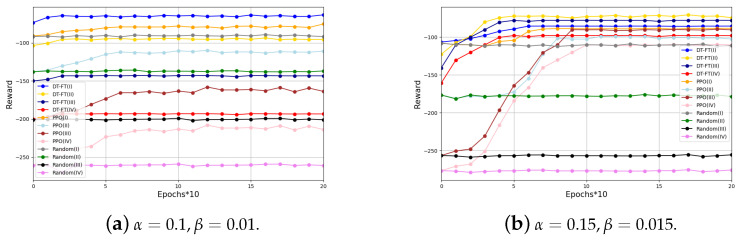
Cumulative reward comparison of DT-FT and PPO in different scenarios.

In Figure 3, we show the performance of DT-FT with PPO and a random selection method in different scenarios for processing new tasks. Figure 3 shows that DT-FT performs better than PPO in handling new tasks in four scenarios. The convergence speed of DT can be increased by up to three times, and the performance can be improved by up to 30%. This is mainly due to the DT’s trajectory learning ability, which allows it to capture the complex internal relationships between states, actions, and rewards. In addition, the DT’s proficiency in utilizing offline training samples allows it to quickly converge to new tasks using only a small number of samples.

**Figure 4:**
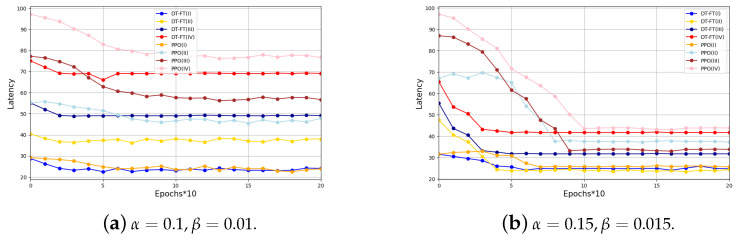
Latency comparison of DT-FT and PPO in different scenarios.

**Figure 5:**
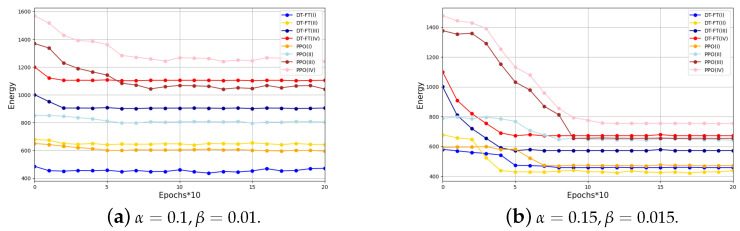
Energy consumption comparisons of DT-FT and PPO in different scenarios.

In Figure 4 and Figure 5, we show the cumulative latency and cumulative energy consumption of PPO and DT-FT when processing a new task in different scenarios. As can be seen in Figure 4, the cumulative delay of DT-FT is smaller than that of PPO in Experiment II, Experiment III, and Experiment IV, except that in Experiment I, the cumulative delay of DT-FT and PPO is almost the same when processing a new task. We analyzed that it may be the fact that the scenarios are simpler in Experiment I, which leads to a reduced possibility of further optimizing the delay. From Figure 5, it can be seen that the energy consumption of DT-FT for processing new tasks is less than that of PPO in different scenarios. In addition, the convergence rate of DT-FT in Figure 4 and Figure 5 is also faster than that of PPO. This further reflects the good generalization and performance improvement of DT.

**Figure 6:**
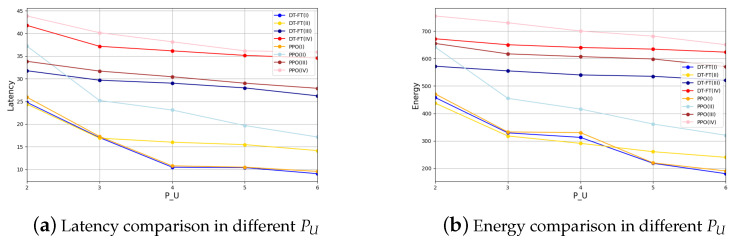
Latency and energy comparison of DT-FT and PPO in different $P_{U}$.

**Figure 7:**
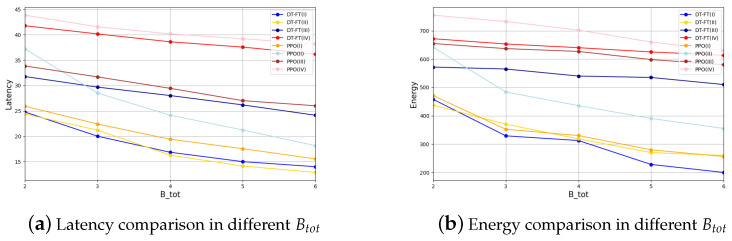
Latency and energy comparison of DT-FT and PPO in different $B_{tot}$.

In Figure 6 and Figure 7, we analyze the case of $P_{U}$ and $B_{tot}$ taking different values. As you can see, in Experiment I, DT and PPO have similar performance. In other cases, DT performs better than PPO. This may be due to the fact that Experiment I is simpler and has less room for optimization. This proves that the performance of DT is not affected by the parameters $P_{U}$ and $B_{tot}$, reflecting the universality of our experimental conclusions.

## 5. Conclusions

In this paper, we investigate resource allocation for data offloading in collaborative LEO-IoT to minimize delay and energy consumption. First, we formulate the resource allocation for data offloading as a multivariate, multiobjective optimization problem. Based on this, the problem is solved using DT. Finally, we conduct a large number of experiments with different configurations, and the results demonstrate that DT outperforms ordinary reinforcement learning algorithms in data offloading. Our work provides an effective solution for the important area of data offloading based on LEO-IoT. In the future, we will consider modifying the structure of the DT to achieve performance improvements and apply it to more complex scenarios.

## Figures and Tables

**Table 1:** Simulation parameters.

Parameter	Value
Total period of time (*T*)	90 s
Total time slots (*W*)	450 m
Total bandwidth for each satellite ($B^{t}ot$)	300 MHz
Total power of each user ($P_{U}$)	3 dBw
Total power of each LEO satellite ($P_{L}$)	12 dBW
Noise spectral density ($N_{0}$)	−174 dBm/Hz
Wave length ($\lambda^{\prime }$)	15 mm
Antenna gain (*G*)	30 dBi

## Data Availability

The data presented in this study are available on request from the corresponding author.

## References

[1] Naik S., Ganesha P. An Architecture for Performance Improvement of IoT Mobile Application Verification Using Statistical Methods. Proceedings of the 2022 IEEE 8th World Forum on Internet of Things (WF-IoT).

[2] Sharbaf M.S. IoT Driving New Business Model, and IoT Security, Privacy, and Awareness Challenges. Proceedings of the 2022 IEEE 8th World Forum on Internet of Things (WF-IoT).

[3] Chen G., Huang R., Tang X., Huang Q. Research on the 5G MEC Co-Construction and Sharing. Proceedings of the 2024 4th International Conference on Neural Networks, Information and Communication (NNICE).

[4] Huang W., Ding Z. (2024). New Insight for Multi-User Hybrid NOMA Offloading Strategies in MEC Networks. IEEE Trans. Veh. Technol..

[5] Ma C., Li J., Wei K., Liu B., Ding M., Yuan L., Han Z., Vincent Poor H. (2023). Trusted AI in Multiagent Systems: An Overview of Privacy and Security for Distributed Learning. Proc. IEEE.

[6] Zeng G., Zhan Y., Xie H. (2023). Channel Allocation for Mega LEO Satellite Constellations in the MEO–LEO Networked Telemetry System. IEEE Internet Things J..

[7] Shi L., Yang F., Wu W., Sun A., Sun Y., Sun T. Load Balancing and Remaining Visible Time Based Handover Algorithm for LEO Satellite Network. Proceedings of the 2022 IEEE 8th International Conference on Computer and Communications (ICCC).

[8] Li J., Shao Y., Wei K., Ding M., Ma C., Shi L., Han Z., Poor H.V. (2022). Blockchain Assisted Decentralized Federated Learning (BLADE-FL): Performance Analysis and Resource Allocation. IEEE Trans. Parallel Distrib. Syst..

[9] Lyu Y., Liu Z., Fan R., Zhan C., Hu H., An J. (2023). Optimal Computation Offloading in Collaborative LEO-IoT Enabled MEC: A Multiagent Deep Reinforcement Learning Approach. IEEE Trans. Green Commun. Netw..

[10] Wei K., Li J., Ding M., Tao M., Poor H.V. (2023). Personalized Federated Learning With Differential Privacy and Convergence Guarantee. IEEE Trans. Inf. Forensics Secur..

[11] Zhang S., Li J., Shi L., Ding M., Nguyen D.C., Tan W., Weng J., Han Z. (2024). Federated Learning in Intelligent Transportation Systems: Recent Applications and Open Problems. IEEE Trans. Intell. Transport. Syst..

[12] Jin L., Wang L., Jin X., Zhu J., Duan K., Li Z. Research on the Application of LEO Satellite in IOT. Proceedings of the 2022 IEEE 2nd International Conference on Electronic Technology, Communication and Information (ICETCI).

[13] Kodheli O., Andrenacci S., Maturo N., Chatzinotas S., Zimmer F. Resource Allocation Approach for Differential Doppler Reduction in NB-IoT over LEO Satellite. Proceedings of the 2018 9th Advanced Satellite Multimedia Systems Conference and the 15th Signal Processing for Space Communications Workshop (ASMS/SPSC).

[14] Gao Z., Liu A., Han C., Liang X. (2021). Max Completion Time Optimization for Internet of Things in LEO Satellite-Terrestrial Integrated Networks. IEEE Internet Things J..

[15] Fang X., Feng W., Wei T., Chen Y., Ge N., Wang C.-X. (2021). 5G Embraces Satellites for 6G Ubiquitous IoT: Basic Models for Integrated Satellite Terrestrial Networks. IEEE Internet Things J..

[16] Zhao X., Yuan Y., Wang A., Zheng T.-X., Lei L. Optimality Analysis and Efficient Scheduling for Massive IoT-LEO Satellite Networks. Proceedings of the 2023 IEEE 24th International Workshop on Signal Processing Advances in Wireless Communications (SPAWC).

[17] Tondo F.A., Souto V.D.P., Alcaraz Lopez O.L., Montejo-Sanchez S., Cespedes S., Souza R.D. (2023). Optimal Traffic Load Allocation for Aloha-Based IoT LEO Constellations. IEEE Sens. J..

[18] Liu Q., Shi L., Sun L., Li J., Ding M., Shu F.S. (2020). Path Planning for UAV-Mounted Mobile Edge Computing with Deep Reinforcement Learning. IEEE Trans. Veh. Technol..

[19] Chai F., Zhang Q., Yao H., Xin X., Gao R., Guizani M. (2023). Joint Multi-Task Offloading and Resource Allocation for Mobile Edge Computing Systems in Satellite IoT. IEEE Trans. Veh. Technol..

[20] Han D., Ye Q., Peng H., Wu W., Wu H., Liao W., Shen X. (2023). Two-Timescale Learning-Based Task Offloading for Remote IoT in Integrated Satellite–Terrestrial Networks. IEEE Internet Things J..

[21] Cao X., Yang B., Shen Y., Yuen C., Zhang Y., Han Z., Poor H.V., Hanzo L. (2023). Edge-Assisted Multi-Layer Offloading Optimization of LEO Satellite-Terrestrial Integrated Networks. IEEE J. Select. Areas Commun..

[22] Chen L., Lu K., Rajeswaran A., Lee K., Grover A., Laskin M., Abbeel P., Srinivas A., Mordatch I. (2024). Decision Transformer: Reinforcement Learning via Sequence Modeling. Proceedings of the 35th International Conference on Neural Information Processing Systems (NIPS ’21).

[23] Zheng Q., Zhang A., Grover A. Online Decision Transformer. Proceedings of the 39th International Conference on Machine Learning Research.

[24] Zhang J., Li J., Wang Z., Shi L., Jin S., Chen W., Poor H.V. (2024). Decision Transformer for Wireless Communications: A New Paradigm of Resource Management. IEEE Wirel. Commun..

[25] Liao H., Zhou Z., Zhao X., Wang Y. (2021). Learning-Based Queue-Aware Task Offloading and Resource Allocation for Space–Air–Ground-Integrated Power IoT. IEEE Internet Things J..

[26] Hu X., Liao X., Liu Z., Liu S., Ding X., Helaoui M., Wang W., Ghannouchi F.M. (2020). Multi-Agent Deep Reinforcement Learning-Based Flexible Satellite Payload for Mobile Terminals. IEEE Trans. Veh. Technol..

[27] Tang Q., Fei Z., Li B., Han Z. (2021). Computation Offloading in LEO Satellite Networks With Hybrid Cloud and Edge Computing. IEEE Internet Things J..

[28] Zhu X., Jiang C., Yin L., Kuang L., Ge N., Lu J. (2018). Cooperative Multigroup Multicast Transmission in Integrated Terrestrial-Satellite Networks. IEEE J. Select. Areas Commun..

[29] Christopoulos D., Chatzinotas S., Ottersten B. (2015). Multicast Multigroup Precoding and User Scheduling for Frame-Based Satellite Communications. IEEE Trans. Wireless Commun..

[30] Li J., Xue K., Wei D.S.L., Liu J., Zhang Y. (2020). Energy Efficiency and Traffic Offloading Optimization in Integrated Satellite/Terrestrial Radio Access Networks. IEEE Trans. Wireless Commun..

[31] Vaswani A., Shazeer N., Parmar N., Uszkoreit J., Jones L., Gomez A.N., Kaiser Ł., Polosukhin I. (2017). Attention Is All You Need. Proceedings of the 31st International Conference on Neural Information Processing Systems (NIPS’17).

[32] Meng L., Wen M., Le C., Li X., Xing D., Zhang W., Wen Y., Zhang H., Wang J., Yang Y. (2023). Offline Pre-Trained Multi-Agent Decision Transformer. Mach. Intell. Res..

[33] Schulman J., Wolski F., Dhariwal P., Radford A., Klimov O. (2017). Proximal Policy Optimization Algorithms. arxiv.

[34] Gu Y., Cheng Y., Chen C.L.P., Wang X. (2022). Proximal Policy Optimization With Policy Feedback. IEEE Trans. Syst. Man Cybern, Syst..

[35] Liu C., Li D. Multi-Agent Proximal Policy Optimization via Non-Fixed Value Clipping. Proceedings of the 2023 IEEE 12th Data Driven Control and Learning Systems Conference (DDCLS).

